# Pedigree-assisted genotype imputation enables cost-effective genomic prediction in *Penaeus vannamei*

**DOI:** 10.1038/s41598-026-47716-y

**Published:** 2026-04-08

**Authors:** Mianyu Liu, Qiang Fu, Ping Wang, Guangfeng Qiang, Kun Luo, Baolong Chen, Sheng Luan, Jie Kong, Pao Xu

**Affiliations:** 1 Wuxi Fisheries College, Nanjing Agricultural University, Wuxi, 214081 China; 2 State Key Laboratory of Mariculture Biobreeding and Sustainable Goods, Yellow Sea Fisheries Research Institute, Chinese Academy of Fishery Sciences, Qingdao, 266071 Shandong China; 3 Hainan Zhongzheng Aquatic Science and Technology Co., Ltd. Dongfang, Hainan, 572633 China

**Keywords:** *Penaeus vannamei*, SNP array, Genotype imputation, Genomic Prediction, Evolution, Genetics

## Abstract

Genomic selection in *Penaeus vannamei* has long been constrained by the high cost of dense genotyping. To address this limitation, we evaluated genotype imputation from a low-density 1 K panel to a medium-density 55 K panel of the “Yellow Sea Array No. 1” and examined its impact on genomic prediction for harvest body weight in *P. vannamei*. A four-generation pedigree including 30 great-grandparents, 39 grandparents, 100 parents, and 608 offspring was genotyped using the 55 K panel. A two-step experimental design was implemented to (i) assess the performance of different imputation algorithms under reference population scenarios with varying proportions of siblings, and (ii) compare six alternative reference population structures incorporating parents, ancestors, and siblings. Genotype imputation using the pedigree-based method FImpute v3.0 consistently achieved higher accuracy than the population-based method Beagle v5.5. Using this pedigree-assisted approach, imputation accuracy increased from 0.73 when only parental genotypes were used to 0.84 with the inclusion of 10% siblings, and subsequently plateaued at 0.87–0.90 when sibling representation reached 20%. Across the six reference population structures, imputation accuracy was primarily driven by the availability of parental genotypes, ranging from 0.50 to 0.56 in the absence of parents to 0.88–0.89 when both parents and ancestral generations were included. Accuracy remained high when both parents were available (0.84–0.87 with siblings; 0.73 without siblings) but declined substantially when only one parent was genotyped (0.65–0.68). Imputation accuracy was positively associated with both minor allele frequency (MAF) and linkage disequilibrium (max r^2^_LD_), with LD exerting the stronger influence. Heritability estimates derived from imputed 55 K genotypes were highly consistent with those obtained from the original 55 K data (0.39 ± 0.14 vs. 0.41 ± 0.14), indicating that genotype imputation did not compromise variance component estimation. In predictive ability analyses, pedigree-based BLUP (PBLUP) achieved higher predictive ability than genomic BLUP (GBLUP) based on the 1 K panel, with predictive abilities of 0.42–0.44 for PBLUP compared with 0.34–0.35 for GBLUP. Using imputed genotypes for genomic prediction further improved predictive ability relative to the true 1 K panel, yielding values ranging from 0.35 to 0.47. Notably, when parental genotypes were included in the reference population, GBLUP based on imputed genotypes surpassed the predictive ability of PBLUP and approached that achieved with the original 55 K genotypes (0.45–0.47). Collectively, these results provide the first empirical evidence that low- to medium-density genotype imputation, combined with pedigree information, can effectively support genomic prediction in *P. vannamei*. This study establishes a cost-efficient and scalable framework for implementing genomic selection in *P. vannamei* and provides a practical reference for the application of genomic selection in other aquaculture species with constrained breeding budgets.

**Supplementary Information:**

The online version contains supplementary material available athttps://doi.org/10.1038/s41598-026-47716-y.

## Introduction

The rapid advancement of genomic technologies has profoundly transformed selective breeding in aquaculture, enabling accurate genetic evaluation and accelerating improvement for economically important traits. Among these innovations, genomic selection (GS) has emerged as a powerful approach that predicts individual genetic merit using genome-wide single nucleotide polymorphism (SNP) data^1^. By integrating genomic information into selection schemes, GS can increase selection accuracy, particularly for traits that require destructive or otherwise costly measurement. In *Penaeus vannamei*, GS has demonstrated improved accuracy in estimating breeding values for key traits, For instance, Lillehammer, et al.^2^ reported that one generation of GS achieved a 13% increase in survival after white spot syndrome virus challenge, Similarly, Liu, et al.^3^ chieved a 10.15% improvement in resistance to acute hepatopancreatic necrosis disease through GS, highlighting the potential of GS to accelerate genetic progress in shrimp breeding.

Despite these encouraging results, the practical implementation of GS in aquaculture remains constrained by the high cost of SNP genotyping, even when using medium-density arrays. Shrimp breeding programs typically manage hundreds or even thousands of full-sib families and the high costs of genotyping each generation make large-scale implementation economically infeasible. In livestock and major aquaculture species, a widely adopted cost-reduction strategy is to genotype most individuals with low-density panels, while genotyping a smaller subset of key individuals or parents with high-density panels^4^. Missing genotypes in low-density data can then be inferred through genotype imputation, which leverages linkage disequilibrium (LD) and pedigree information to statistically recover missing markers^5–8^. This strategy of combining low-density panels genotyping with imputation has been successfully reported in species such as Atlantic salmon, rainbow trout, and carp, where imputation from 0.5 to 5 K to 50 K panels achieved accuracies exceeding 0.90 and maintained comparable genomic prediction accuracy to true high-density data^9–12^.

However, similar systematic evaluations of SNP array-based imputation are virtually absent in crustaceans, despite the growing interest in genomic applications for shrimp breeding. This knowledge gap is primarily due to two key constraints. First, for many prominent crustaceans, there is still a lack of paired medium- and low-density SNP panels with shared markers, which are essential for evaluating SNP array-based imputation performance. Even for *P. vannamei*, paired SNP array has only recently become available. Second, species in the family Penaeidea have an average genome size of 2.64 pg^13^. Furthermore, the genome of *P. vannamei* is characterized by an extremely high repetitive content, with approximately 78% consisting of repetitive sequences^14^. These features have historically complicated genome assembly and may contribute to rapid LD decay, which in turn limits the effectiveness of LD-based genotype imputation. Given these challenges, effective imputation in *P. vannamei* requires alternative strategies. One such approach is the incorporation of pedigree information to better construct haplotypes, as it can help overcome the limitations of low-density SNP arrays and improve imputation accuracy^15^.

The recent development of the “Yellow Sea Array No. 1” SNP array series provides a unique opportunity to address this gap. Designed specifically for *P. vannamei*, this series includes a medium-density 55 K panel together with a hierarchically derived low-density 1 K panel, providing a practical framework for genotype imputation between SNP arrays. Using genotypic data from individuals with recorded pedigrees spanning four generations, we conduct the first systematic assessment of imputation accuracy for low-density SNP arrays in *P. vannamei* and evaluate the downstream impact of imputed genotypes on genomic prediction. Specifically, this study aims to: (1) compare the performance of different imputation algorithms under reference population scenarios with varying proportions of siblings; (2) evaluate the effects of varying pedigree-based reference scenarios on imputation performance; and (3) compare the predictive ability of imputed genotypes with those obtained using the true 1 K and 55 K panels for harvest body weight. By quantifying both imputation and prediction accuracy across multiple reference configurations, this study provides empirical reference data for evaluating genotype imputation in *P. vannamei* breeding programs.

## Materials and methods

### The high-density and low-density panels

This study employed two SNP panels for genotype imputation in *P. vannamei*: a high-density 55 K SNP panel and a low-density 1 K SNP panel of “Yellow sea Array No.1” series. The development of the 55 K SNP array was based on whole-genome resequencing data from 433 individuals representing eight distinct populations with broad genetic diversity, including commercial strains and wild populations from Ecuador. Through an updated iteration process, we ultimately obtained an optimized set of 56,214 SNPs demonstrating comprehensive genome-wide coverage and high genotyping accuracy. Building upon this 55 K array as the foundation, our research team subsequently developed lower-density arrays: the 1 K array was constructed by carefully selecting 1,140 highly informative and evenly distributed SNPs from the 55 K array using genotyping data from 2,330 samples optimized through the MOLO algorithm^16^. The MOLO algorithm was applied to select 1,140 SNPs by maximizing locus-average Shannon entropy ($\:\stackrel{-}{{H}_{L}}$) while ensuring even genomic distribution and inclusion of mandatory loci. The weights for minor allele frequency (MAF) based informativeness (w1) and genomic uniformity (w2) were both set to 0.5, reflecting equal prioritization of marker information content and genome-wide coverage. In the current study, the 55 K array served as our high-density reference panel, while the 1 K array functioned as the low-density panel for downstream applications.

### Phenotype and genotype

The shrimp used in this study were sourced from the Zhongzheng Aquatic Science and Technology Co., Ltd. (Dongfang, Hainan Province, China). A base population (G0) was established in 2020, with subsequent selection generations (G1 to G5) maintained as closed populations. From the G5 generation, a total of 50 full-sib families were selected for the growth performance evaluation. At post-larval stage 20, 30 individuals per family were randomly selected and communally reared in a single controlled-environment indoor concrete tank (6.4 m^3^). Initial body lengths were recorded for each family before communal stocking. After the 3 months growth period, the harvested body weights were measured and tissue samples were collected.

A subset of individuals involved in the growth test and their ancestors were genotyped using the “Yellow Sea Array No. 1” 55 K SNP panel. Specifically, a random subset comprising 50% of the G5 test individuals (608 individuals) was selected for genotyping. In addition, all 100 parents (50 sires and 50 dams) from the G4 generation of the G5 test population were genotyped, together with 39 grandparents from the G3 generation and 30 great-grandparents from the G2 generation, resulting in a total of 777 genotyped individuals. Based on these genotypic data, pedigree relationships were subsequently reconstructed using COLONY software^17^. For the G5 generation, all individuals were successfully assigned to their corresponding full-sib families, and clear sibship structures were inferred for all 50 families. Before quality control, all SNPs were aligned to an unpublished genome (Genbank accession: JBRAUT000000000), which has a total size of 2.43 Gb, a contig N50 of 35.77 Mb, and comprises 49 scaffolds. The 55 K SNPs were distributed across all scaffolds with relatively uniform coverage, and the 1 K SNPs show a similar distribution pattern, reflecting the optimization strategy applied during panel design (Supplementary Fig. 1). Quality control was performed using PLINK v1.9 software^18^(https://www.cog-genomics.org/plink2/), removing low-quality SNPs with minor allele frequency (MAF) below 0.05, call rate below 0.9, or more than two alleles, as well as samples with genotype call rates below 0.8. After quality control, 41,013 SNPs and 777 individuals remained for subsequent analysis. To construct the low-density dataset, SNPs that overlapped with the “Yellow Sea Array No.1” 1 K panel were selected from the quality-controlled genotyped data, resulting in a final set of 1,022 SNPs for downstream analysis.

### Imputation scenarios

This study employed a two-step experimental design to systematically evaluate genotype imputation accuracy under different reference population configurations in *P. vannamei*. Step 1 assessed the effect of different imputation algorithms under reference population scenarios with varying proportions of siblings, whereas Step 2 evaluated the impact of reference population composition under scenarios relevant to commercial breeding programs.

In Step 1, ten sibling inclusion proportions ranging from 0% to 90% at 10% intervals were evaluated. For each proportion, 20 replicates were generated by standardized random sampling from 50 full-sib families. In each replicate, the specified proportion of siblings together with all corresponding parents constituted the reference population, while the remaining siblings served as validation individuals. For validation individuals, only genotypes from the 1 K SNP panel were retained, and all remaining genotypes from the 55 K SNP panel were masked. Genotype imputation was performed independently using FImpute v3.0^15^ (https://www.uoguelph.ca/ovc/pathobiology/people/faculty/mehdi-sargolzaei) and Beagle v5.5^19^ (https://faculty.washington.edu/browning/beagle/beagle.html).

Imputation accuracy was evaluated using two metrics: allelic dosage correlation (DR) and genotype concordance rate (GC)^20,21^. Genotypes were coded as 0, 1, 2 according to allele counts. For all validation individuals and imputed genotypes, the observed true genotypes were derived from the original 55 K SNP array dataset, and the corresponding imputed genotypes were obtained from the imputation output. To facilitate calculation, all observed true genotypes were concatenated into a single vector * tg*, and all imputed genotypes were concatenated into a corresponding vector * ig*. The two vectors follow exactly the same order across individuals and SNPs. DR was calculated as the Pearson correlation coefficient between the two genotype vectors:

$$\:DR=\:\frac{\sum\:_{i=1}^{n}({tg}_{i}-\stackrel{-}{tg})({ig}_{i}-\stackrel{-}{ig})}{\sqrt{\sum\:_{i=1}^{n}{(t{g}_{i}-\stackrel{-}{tg})}^{2}\sum\:_{i=1}^{n}{({ig}_{i}-\stackrel{-}{ig})}^{2}}}$$

Where $\:{tg}_{i}$ and $\:{ig}_{i}$ the observed true and imputed genotype for the $\:{i}^{th}$ genotype, respectively; $\:\stackrel{-}{tg}$ and $\:\stackrel{-}{ig}$ are the mean values of the observed true and imputed genotype vectors, and * n* is the total number of genotype calls evaluated, equal to the number of validation individuals multiplied by the number of imputed SNPs. Genotype concordance rate (GC) was defined as the proportion of identical genotype calls between the two vectors:

$$\:GC=\frac{1}{n}{\sum\:}_{i=1}^{n}I({tg}_{i}={ig}_{i})$$

where $\:I(\cdot\:)$ is an indicator function that equals 1 when the imputed genotype matches the observed true genotype and 0 otherwise.

Results from Step 1 indicated that imputation accuracy reached a plateau at a sibling inclusion proportion of approximately 20%, beyond which additional siblings yielded negligible improvements (Δr < 0.01). Although the 10% sibling inclusion level resulted in a slight reduction in accuracy compared with the 20% level, it represents a more cost-effective alternative in practical applications. Therefore, both the 10% and 20% sibling inclusion levels were selected for evaluation in Step 2.

In Step 2, six reference population scenarios were evaluated for genotype imputation. In Scenario 1 (S1), the reference population consisted of genotyped individuals from all available ancestral generations (G2-G4), including great-grandparents, grandparents, and parents, as well as G5 offspring. In Scenario 2 (S2), the reference population included genotyped parents and offspring. In Scenario 3 (S3), the reference population consisted only of genotyped parents. Scenarios 4 (S4) and 5 (S5) were identical to S2, except that genotype from dams and sires, respectively, were excluded from the reference population. In Scenario 6 (S6), the reference population consisted only of genotyped offspring.

For S1, S2, S4, S5, and S6, reference populations were constructed by including 10% or 20% of siblings, as defined in Step 1 (corresponding to 60 and 123 individuals, respectively), while the remaining siblings (548 and 485 individuals) were assigned to the validation sets. In contrast, S3 employed a reference population containing no siblings but was validated against the same sibling sets. As in Step 1, validation individuals were processed by retaining only the 1 K panel genotypes, with all other 55 K SNPs set to missing. Each scenario was replicated 20 times by independently resampling the sibling individuals from the 50 full-sib families according to the predefined proportion, while the parental and ancestral individuals included in each scenario remained fixed. All genotype imputation was performed using FImpute v3.0^15^, which was selected based on its superior accuracy observed in Step 1. Imputation accuracy was evaluated using the same metrics as in Step 1.

### Evaluating accuracy across MAF and LD

PopLDdecay v3.42^22^ (https://github.com/BGI-shenzhen/PopLDdecay/releases) was used to calculate the LD decay based on the 55 K SNP dataset, and the resulting LD decay curve was used to represent the strength and decay of LD with increasing physical distance between markers in the studied population. The imputation accuracy is influenced by the MAF of the variant and its LD with genotyped variants. To systematically evaluate the effects of these factors, we analysed the impact of MAF and LD on accuracy across the six imputation scenarios and the two different sibling proportions included in the imputation step 2. Specifically, for each imputed SNP, the genotyped SNP in the region with the strongest LD was selected to define its maximum r^2^_LD_ value (denoted as max r^2^_LD_). All LD calculations were performed using PLINK v1.9 software^18^. In all scenarios, the MAF and max r^2^_LD_ were then partitioned into bins with increments of 0.05 and 0.1, respectively, and the mean impute accuracy was calculated for each bin.

### Genomic predictions

To evaluate the impact of genotype imputation errors on genomic selection for harvest body weight in *P. vannamei*, we performed validation using the genomic best linear unbiased prediction (GBLUP) model using two genomic relationship matrices (G matrices) for comparison: the $\:{G}_{raw}$ matrix constructed from the observed true genotypes of all genotyped individuals. In contrast, the $\:{G}_{impute}$ matrix was constructed using imputed 55 K genotypes for the 608 offspring, which were imputed using the genotypes of all parents as the reference population, together with the observed true genotypes of all parental individuals.

Variance components for harvest body weight were estimated using ASReml v4.2 software^23^(https://asreml.kb.vsni.co.uk/asreml-r-4-download-success/?site_reference=VS19AAN5) under GBLUP. The full model was as follows:

1 $$\:\begin{array}{c}{y}_{ijk}=\mu\:+{Sex}_{i}+{\beta\:BL}_{j}\left({Sex}_{i}\right)+{a}_{j}+{c}_{k}{+e}_{ijk}\end{array}$$

where $\:{y}_{ijk}$ is the observed harvest body weight of the *j*^th^ individual; $\:\mu\:$ is the overall mean; $\:{Sex}_{i}$ is the fixed effect of the *i*^th^ sex; $\:{BL}_{j}\left({Sex}_{i}\right)$ is a linear covariate of the initial body length of the *j*^th^ individual nested within the *i*^th^ sex, $\:\beta\:$ is the regression coefficient of the covariate; $\:{a}_{j}$ is the additive genetic effect of the *j*^th^ individual assumed to be normally distributed, $\:{a}_{j}\sim\left(0,{G\sigma\:}_{\boldsymbol{a}}^{2}\right)$, in which * G* represents either $\:{G}_{raw}$ or $\:{G}_{impute}$, and $\:{\sigma\:}_{a}^{2}$ is the additive genetic variance; $\:{c}_{k}$ is the full-sib effect for the *k*^th^ full-sib family, which is due to separate rearing of the full-sib families before communal rearing and one-quarter of the non-additive genetic effect (dominance) common to full-sibs, $\:{c}_{k}\sim(0,{I\sigma\:}_{c}^{2})$, *I* is the identity matrix, $\:{\sigma\:}_{c}^{2}$ is the variance of the full-sib effect; and $\:{e}_{ijk}$ is the random residual error of the *j*^th^ individual, $\:\mathrm{e}\sim(0,{I\sigma\:}_{e}^{2})$, where $\:{\sigma\:}_{e}^{2}$ is the residual variance. The phenotypic variance $\:\left({\sigma\:}_{p}^{2}\right)$ was calculated as $\:{\sigma\:}_{p}^{2}=\:{\sigma\:}_{a}^{2}+{\sigma\:}_{c}^{2}+\:{\sigma\:}_{e}^{2}$. The narrow-sense heritability was estimated as $\:{h}^{2}={\sigma\:}_{a}^{2}/{\sigma\:}_{p}^{2}$.

In the Step 2 imputation trials, 10% and 20% of siblings were assigned to the reference populations, thereby leaving the remaining siblings as two independent validation sets comprising 548 (Set-548) and 485 (Set-485) individuals, respectively. These validation sets were subsequently used to assess genomic predictive ability.

To evaluate the predictive performance of each imputation scenario, both pedigree-based BLUP (PBLUP) and GBLUP were assessed using the same 10-fold cross-validation scheme. For each validation set, individuals were randomly partitioned into 10 equal and mutually exclusive subsets. In each iteration, one subset was designated as the validation set, with phenotypic records masked, while the remaining nine subsets were used as the training set. In the PBLUP analyses, estimated breeding values (EBVs) for individuals in the validation set were predicted using the pedigree-based model, with the same model structure as described above, except that the pedigree-based relationship matrix was used. In the GBLUP analyses, genomic estimated breeding values (GEBVs) were predicted using the same model, with genomic relationship matrices constructed from true or imputed genotypes, depending on the scenario.

Predictive ability was calculated as the Pearson correlation between predicted breeding values (EBVs or GEBVs) and observed phenotypes:

$$\:Predictive\:ability=cor\:\left(BV,y\right)$$

where * BV* denotes EBVs for PBLUP or GEBVs for GBLUP. To ensure robustness, the 10-fold cross-validation process was repeated 10 times, and the final predictive ability estimates were averaged across all folds and repetitions.

## Result

### Descriptive statistics

Table 1 summarizes the descriptive statistics for harvest body weight in the G5 generation. The number of individuals successfully assigned to each full-sib family ranged from 5 to 36, with an average of 12. The average assigned family size (12 individuals) was lower than the initial 30 individuals stocked per family, reflecting normal mortality during grow-out and the genotyping strategy in which approximately 50% of individuals were randomly selected. One family contained slightly more than 30 assigned individuals (36 individuals), likely due to minor counting inaccuracies during the early communal rearing phase. Females showed a higher mean body weight (18.05 g) than males (17.22 g). The overall weight range in the population was 3.8 g to 31.5 g.

**Table 1:** Descriptive statistics of harvest body weight in the G5 generation of *Penaeus vannamei*.

Sex	Number of individuals	Harvest body weight				
		Mean (g)	Minimum (g)	Maximum (g)	Standard deviation (g)	Coefficient of variation (%)
Male	299	17.22	3.8	29.3	4.73	27.46
Female	309	18.05	3.9	31.5	4.53	25.11
All	608	17.64	3.8	31.5	4.64	26.32

### Accuracy of genotype imputation

**Fig. 1:**
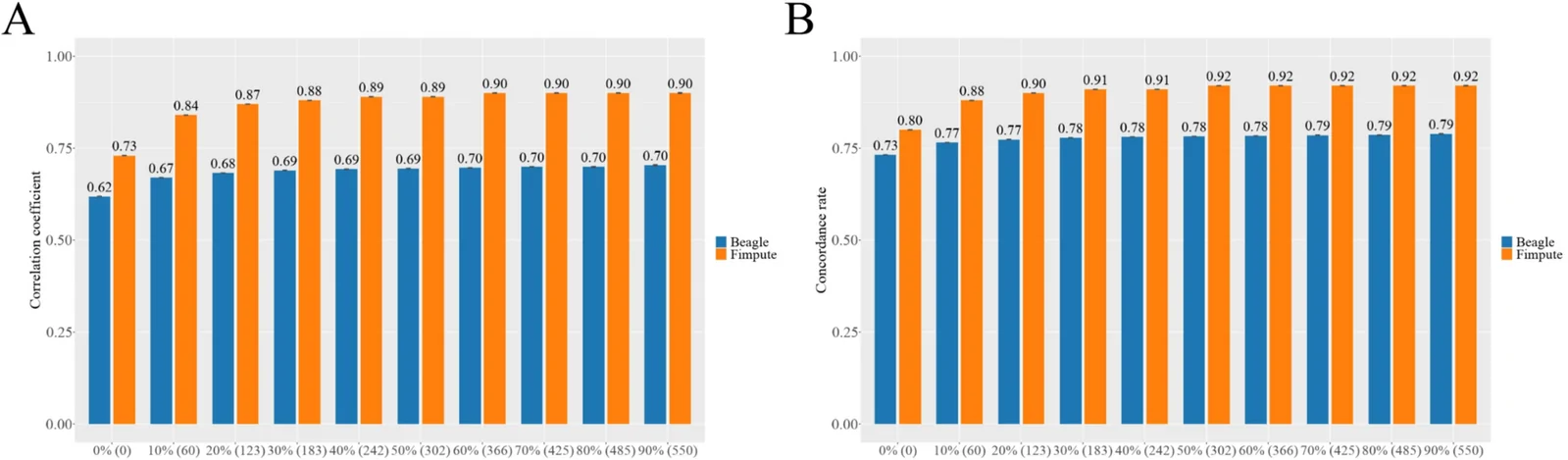
Genotype imputation accuracy using FImpute and Beagle in *Penaeus vannamei* under varying proportions of siblings in the reference population. (**A**) Imputation accuracy measured by Pearson correlation coefficient. (**B**) Imputation accuracy measured by genotype concordance rate.

Figure 1 illustrates the genotype imputation accuracy achieved by Beagle and FImpute across varying sibling proportions in the reference population for *P. vannamei*. Across all tested scenarios, FImpute demonstrated significantly higher imputation accuracy than Beagle, achieving a mean DR of 0.87 compared to 0.68 for Beagle, and a mean GC of 0.90 compared to 0.78. Focusing on FImpute, imputation accuracy increased substantially with the inclusion of siblings in the reference population. When only parental genotypes were included in the reference population, DR was 0.73, whereas inclusion of just 10% of siblings increased DR to 0.84. Increasing the sibling proportion to 20% led to a modest increase in DR to 0.87, beyond which further increases in sibling proportion resulted in only limited improvements. A similar trend was observed for GC, which increased from 0.80 to 0.88 and then to 0.92 across the same sibling inclusion levels.

As shown in Table 2, genotype imputation accuracy was primarily driven by parental information, with sibling genotypes providing additional improvements. Under scenarios that included sibling genotypes, incorporating more extensive parental information improved imputation accuracy. Specifically, S1 achieved higher accuracy than S2 (S1: DR = 0.88–0.89, GC = 0.91; S2: DR = 0.84–0.87, GC = 0.88–0.90), indicating that pedigree tracing to include ancestral parental generations enhances imputation performance. Under the same condition of sibling inclusion, having genotypes from both parents yielded higher accuracy than having only one parental type. S2 consistently outperformed S4 and S5, where either dam or sire genotypes were excluded (S4/S5: DR = 0.65–0.68; GC = 0.74–0.76). In the absence of sibling genotypes, parental information remained crucial. S3 achieved substantially higher accuracy than S6 (S3: DR = 0.73, GC = 0.80; S6: DR = 0.50–0.56, GC = 0.66–0.70), indicating that parental genotypes provide more informative anchors for imputation than sibling genotypes alone. Finally, across all scenarios that included siblings (S1, S2, S4, S5, and S6), increasing the sibling proportion from 10% to 20% led to consistent but modest improvements in both metrics, with mean R increasing from 0.71 to 0.74 and mean GC increasing from 0.78 to 0.81.

**Table 2:** Accuracy of genotype imputation from 1 K to 55 K SNPs in *Penaeus vannamei* under different Scenarios with varying sizes of sibs in reference and validation sets.

Scenario	Great-grandparental					Validation Sibs	DR	GC
	Reference parents	Grandparental	Sires	Dams	Reference Sibs			
Scenario 1	30	39	50	50	60	548	0.88 ± 0.0012	0.91 ± 0.0010
					123	485	0.89 ± 0.0004	0.91 ± 0.0003
Scenario 2	0	0	50	50	60	548	0.84 ± 0.0017	0.88 ± 0.0013
					123	485	0.87 ± 0.0007	0.90 ± 0.0006
Scenario 3	0	0	50	50	0	548	0.73 ± 0.0007	0.80 ± 0.0005
					0	485	0.73 ± 0.0007	0.80 ± 0.0005
Scenario 4	0	0	50	0	60	548	0.65 ± 0.0027	0.74 ± 0.0019
					123	485	0.68 ± 0.0016	0.76 ± 0.0012
Scenario 5	0	0	0	50	60	548	0.65 ± 0.0024	0.74 ± 0.0021
					123	485	0.68 ± 0.0021	0.76 ± 0.0015
Scenario 6	0	0	0	0	60	548	0.50 ± 0.0047	0.66 ± 0.0029
					123	485	0.56 ± 0.0016	0.70 ± 0.0018

### Factors that influence imputation accuracy

**Fig. 2:**
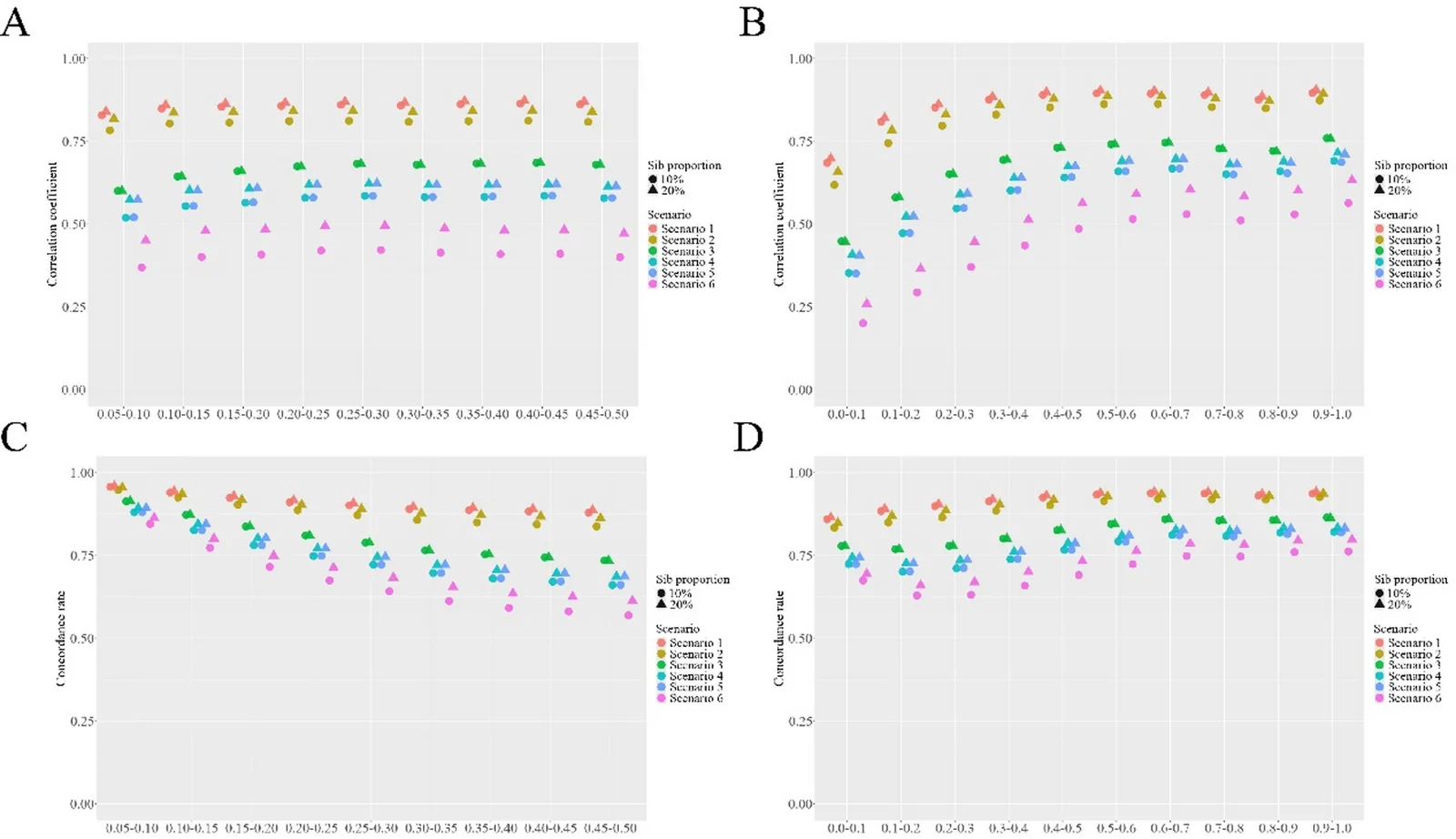
Relationships between genotype imputation accuracy and variant properties under different reference population scenarios in *Penaeus vannamei.* (**A, B**) Pearson correlation coefficient plotted against MAF and max r²_LD_, respectively. (**C, D**) Genotype concordance rate plotted against MAF and max r²_LD_, respectively.

LD decayed rapidly in the studied population (Supplementary Fig. 2). At distances greater than 1 kb, the mean r²_LD_ decreased to approximately 0.12. Figure 2 shows genotype imputation accuracy across different reference population scenarios and sibling proportions under varying minor allele frequency (MAF) and maximum linkage disequilibrium (max r²_LD_) bins. With increasing MAF, the DR increased consistently across all scenarios (Fig. 2A), whereas the GC showed a decreasing trend (Fig. 2C). By comparison, both DR and GC increased with increasing max r²_LD_ (Fig. 2B and D). The difference in imputation accuracy between the 10% and 20% sibling reference proportions was relatively small in scenarios that included parental genotypes. In scenarios that relied exclusively on sibling genotypes, a larger difference between the two sibling proportions was observed. Across all conditions, the 20% sibling reference proportion consistently resulted in higher imputation accuracy than the 10% proportion.

### Variance component and heritability

Table 3 presents the genetic parameter estimates for harvest body weight derived from both the original and the imputed genomic relationship matrices. The heritability for harvest body weight was estimated as 0.41 ± 0.14 using the original 55 K SNP matrix. When using the imputed 55 K SNP matrix, the estimated heritability was 0.39 ± 0.14. The remarkable consistency between these two estimates (0.41 vs. 0.39) demonstrates that genotype imputation had a negligible impact on the heritability estimation. The heritability estimated by GBLUP was significantly higher than that by PBLUP, with a smaller standard error, indicating a more accurate estimation of heritability.

**Table 3:** Variance component and heritability estimate for harvest body weight in *Penaeus vannamei*.

Method	$\:{\boldsymbol{\sigma\:}}_{\boldsymbol{p}}^{2}$	$\:{\boldsymbol{\sigma\:}}_{\boldsymbol{a}}^{2}$	$\:{\boldsymbol{\sigma\:}}_{\boldsymbol{c}}^{2}$	$\:{\boldsymbol{\sigma\:}}_{\boldsymbol{e}}^{2}$	$\:{\boldsymbol{h}}^{2}$	$\:{\boldsymbol{c}}^{2}$
PBLUP	21.20 ± 1.79	4.33 ± 2.94	4.01 ± 6.10	12.86 ± 3.17	0.19 ± 0.28	0.20 ± 0.14
GBLUP-55 K	21.44 ± 1.79	8.77 ± 3.15	2.61 ± 1.69	10.06 ± 1.70	0.41 ± 0.14	0.12 ± 0.08
GBLUP-Impute	21.62 ± 1.83	8.49 ± 3.33	2.60 ± 1.75	10.52 ± 1.71	0.39 ± 0.14	0.12 ± 0.07

$\:{\sigma\:}_{p}^{2}$: phenotypic variance: $\:{\sigma\:}_{a}^{2}$: additive genetic variance; $\:{\sigma\:}_{c}^{2}$: common environmental variance; $\:{\sigma\:}_{e}^{2}$: residual variance; $\:{h}^{2}$: heritability; $\:{c}^{2}$: common environmental coefficient.

### Accuracy of genomic predictions using imputed genotypes

**Fig. 3:**
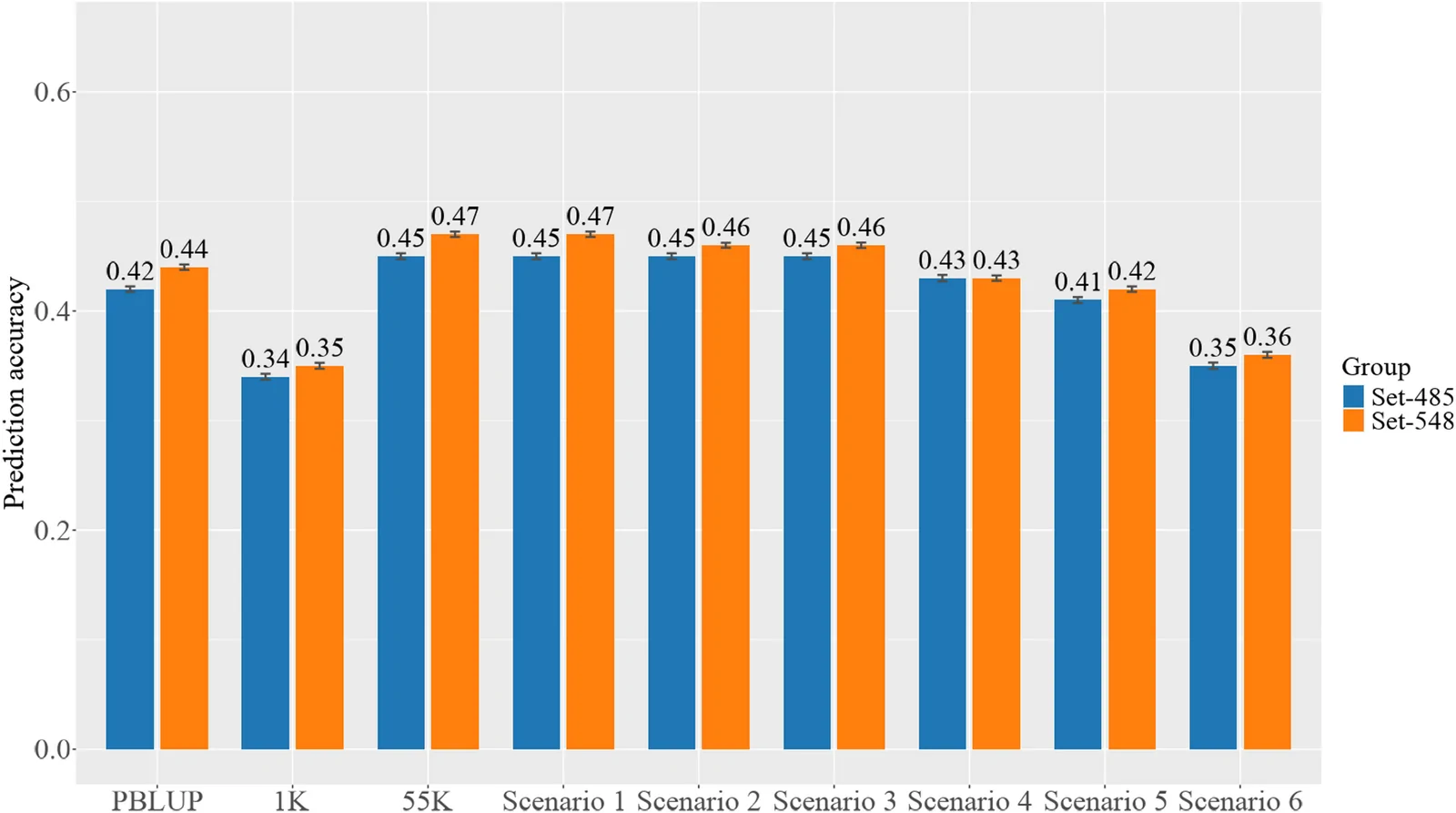
Comparison of predictive ability for body weight between PBLUP and GBLUP using true and imputed SNP panels in *Penaeus vannamei*.

Figure 3 compares the predictive ability for harvest body weight obtained using PBLUP and GBLUP with true (1 K and 55 K) and imputed SNP genotypes under different imputation scenarios in *P. vannamei*. Across both validation sets, PBLUP consistently showed higher predictive ability than GBLUP based on the true 1 K panel, with predictive abilities of 0.42 and 0.44 for Set-485 and Set-548, respectively, compared with 0.34 and 0.35 for the true 1 K panel. Incorporation of high-density genotypes using the true 55 K panel further improved predictive ability beyond PBLUP, reaching 0.45 in Set-485 and 0.47 in Set-548. When imputed genotypes were used in GBLUP, all imputation scenarios resulted in higher predictive ability than the true 1 K panel, demonstrating that genotype imputation substantially enhanced the utility of low-density genotypes for genomic prediction. S1-S3 achieved predictive abilities that exceeded the PBLUP baseline and were comparable to those obtained with the true 55 K panel, with values ranging from 0.45 to 0.47 across both validation sets. In contrast, S4-S6 showed more limited gains, remaining below the PBLUP baseline despite outperforming the true 1 K panel, with prediction accuracies ranging from 0.36 to 0.43.

## Discussion

This study presents the first comprehensive validation of SNP array-based genotype imputation and post-imputation genomic prediction in a crustacean species. By leveraging paired 55 K and 1 K SNP arrays from the “Yellow Sea Array No. 1” in combination with genotyping data spanning four generations, this study demonstrates a scalable and accurate genotype imputation framework for *P. vannamei*, which lowers the cost of GS and enables its application to large breeding populations and other penaeid species.

### Impute accuracy of 1 K panel

The significantly higher imputation accuracy achieved by FImpute compared to Beagle underscores the advantage of pedigree-based approaches in *P. vannamei*. The current version of Beagle does not incorporate pedigree relationships and relies solely on population LD for imputation. In contrast, FImpute utilises pedigree information for constructing haplotypes and imputing unknown genotypes^15^. Pedigree information enables more accurate tracing of shared haplotype segments between reference and target individuals before imputation, leading to substantially improved genotype imputation in *P. vannamei*^24^. Furthermore, imputation accuracy increased with the number of siblings included in the imputation group, a pattern consistent with findings in livestock^25,26^. This can be attributed to the expanded reference population comprising highly related individuals, which enhances shared haplotype information^27^. However, beyond 20% sibling inclusion, the marginal improvement in accuracy diminished considerably, suggesting a practical threshold for cost-effective breeding programs. This is a particularly relevant consideration in aquaculture, where full-sib families often consist of hundreds of individuals.

During the evaluation of the impact of different genotyping strategies on imputation accuracy, it was found that the reference population comprising multi-generational parents (S1) along with siblings achieved the highest genotype imputation accuracy, outperforming the group consisting solely of parents and siblings (S2). This trend aligns with findings in dairy cattle^15^, indicating that increasing the generational depth of genotyped parents contributes to enhancing imputation accuracy. The completeness of parental genotype information is a critical factor influencing imputation efficacy. Under the S2 with complete parental information, imputation accuracy was significantly higher than in any scenario involving only a single parent (S4 and S5), demonstrating that obtaining genotypes from both parents more effectively supports accurate haplotype resolution. These findings are consistent with conclusions from pig studies^28^, which report a positive correlation between the completeness of genotyped parents and imputation accuracy. The comparable imputation accuracy between paternal-only (S4) and maternal-only (S5) scenarios underscore equal parental contributions under our balanced family design. This finding contrasts with the paternal bias reported in rainbow trout, where sires have more offspring^12^. Together, these results establish that family structure is a critical factor affecting the performance of single-parent imputation. Notably, under conditions of similar reference population sizes, the imputation accuracy of S3 (parents-only) was significantly higher than that of S6 (siblings-only), further confirming the fundamental role of parental information in haplotype resolution. This phenomenon has also been observed in rainbow trout studies^11,12^. Finally, across all experimental scenarios, as the sibling reference set expanded from 60 to 123 individuals, accuracy consistently improved, reinforcing the positive correlation between reference population size and imputation reliability.

### Factors Influencing Imputation Accuracy

This study systematically analyzed the effects of MAF and max r^2^_LD_ on genotype imputation accuracy across different imputation scenarios and varying proportions of siblings in the reference population. Previous genotype imputation studies in four aquatic species have reported generally poor DR for low-frequency variants (MAF < 0.1)^29^, and similar patterns were observed in the present study. SNPs with low MAF (0.05–0.10) consistently showed lower DR than high-frequency loci across all imputation scenarios (Fig. 2A), and DR improved steadily as MAF increased. In contrast, GC exhibited an opposite trend with respect to MAF (Fig. 2C). At low MAF, most individuals carry the major homozygous genotype. Under such conditions, even a simple imputation that assigns the major genotype can produce a high GC, potentially inflating the apparent imputation accuracy. In contrast, DR is more sensitive to imputation errors involving rare alleles^30^. This metric-dependent pattern is consistent with observations reported in sheep populations^31,32^.

Compared with MAF, LD exerted a more pronounced and consistent influence on imputation performance. Across all scenarios, both DR and GC increased monotonically with increasing max r^2^_LD_ (Fig. 2B and D). Higher LD reflects stronger local haplotype structure and more informative neighboring markers, thereby providing a more reliable genetic basis for genotype imputation^33^. Although the population exhibits rapid LD decay, which limits the accuracy of imputation in low-LD regions, FImpute algorithm leverages pedigree information to prioritize haplotypes from the longest to the shortest, thereby compensating for the limited long-range LD^15^. As a result, even under relatively low background LD, high imputation accuracy can be maintained when complete parental genotypes are available.

### Predictive ability and cost efficiency

In this study, genomic prediction using GBLUP with imputed genotypes consistently showed higher predictive ability than that based on the true 1 K panel. Similar improvements in genomic prediction following genotype imputation have been reported in other aquaculture species^9–11,29,34^. However, the predictive benefit of genotype imputation was strongly dependent on the composition of the reference population. Only scenarios incorporating comprehensive parental information (S1-S3) achieved predictive abilities that exceeded the predictive ability of PBLUP and approached those obtained with the true 55 K panel. In contrast, scenarios with incomplete or missing parental information (S4-S6) showed substantially reduced predictive ability, remaining below the predictive ability of PBLUP. These results indicate that parental genotypes play a decisive role in enabling imputed marker data to translate into effective gains in genomic prediction, whereas sibling information alone is insufficient to fully compensate for the absence of parental information.

Beyond prediction performance, consistency in genetic parameter estimation further supports the reliability of genotype imputation. In the present study, heritability estimates derived from the imputed genomic relationship matrix were highly consistent with those obtained using the true 55 K genotypes. Similar results have been reported in Japanese Black cattle, where parental-based imputation produced genomic relationship matrices and genetic parameter estimates comparable to those derived from high-density genotypes^35^. Together, these findings suggest that accurate reconstruction of genomic relationships using parental information alone can be sufficient to preserve key genetic parameters when dense genotyping of all individuals is not feasible.

From an application perspective, the parent-only strategy (S3) represents the most cost-effective balance between predictive performance and genotyping expense. Unlike previous studies relying on simulated low-density panels^29^, this study developed and implemented an actual 1 K low-density SNP panel specifically designed for *P. vannamei*. Based on the GBTS technology platform, the per-sample genotyping cost of the 55 K panel is approximately USD 16–20, whereas the cost of the 1 K panel is only USD 5–6. As shown in Table 4, in this study, by genotyping parents with the 55 K panel and imputing offspring genotypes from the 1 K panel, total genotyping costs can be reduced by more than 60% compared with full-population 55 K genotyping. Paired t-tests across replicates indicated that the predictive ability of S3 was not significantly different from S1 and S2 (*P* > 0.05). These results confirm that S3 maintains comparable predictive performance while substantially reducing genotyping costs. This strategy provides a practical and economically feasible solution for large-scale implementation of genomic selection in shrimp.

**Table 4:** Estimated genotyping costs under different imputation Scenarios in *Penaeus vannamei*.

Scenario	55 K Genotyped (*n*)	1 K Genotyped (*n*)	Estimated Total Cost ($)	Cost Reduction vs. Full 55 K (%)
Full 55 K	708	0	14,160	-
S1	229	548	7868	44%
S2	160	548	6488	54%
S3	100	548	5288	63%
S4/S5	110	548	5488	61%
S6	60	548	4488	68%

## Conclusion

This study establishes the first validated framework for SNP array-based imputation and post-imputation genomic prediction in *P. vannamei*. By leveraging high-contiguity genome assemblies and pedigree-assisted algorithms, accurate and cost-efficient genomic selection can be achieved in *P. vannamei*. Using only parental genotypes, comparable prediction performance to full-density genotyping is attainable at a fraction of the cost. These results provide both methodological innovation and practical value, paving the way for economical, high-throughput genomic selection in shrimp breeding programs.

## Supplementary Information

Below is the link to the electronic supplementary material.

## Data Availability

Sequencing data generated for this project have been deposited in the Genome Sequence Archive (GSA) at the China National Center for Bioinformation (CNCB) under accession number CRA062166.
