# Supplementary figures and images for: Pedigree-assisted genotype imputation enables cost-effective genomic prediction in Penaeus vannamei
Source: Sci Rep. 2026 Apr 8;16:16656. doi: 10.1038/s41598-026-47716-y (PMC13219476; doi:10.1038/s41598-026-47716-y)

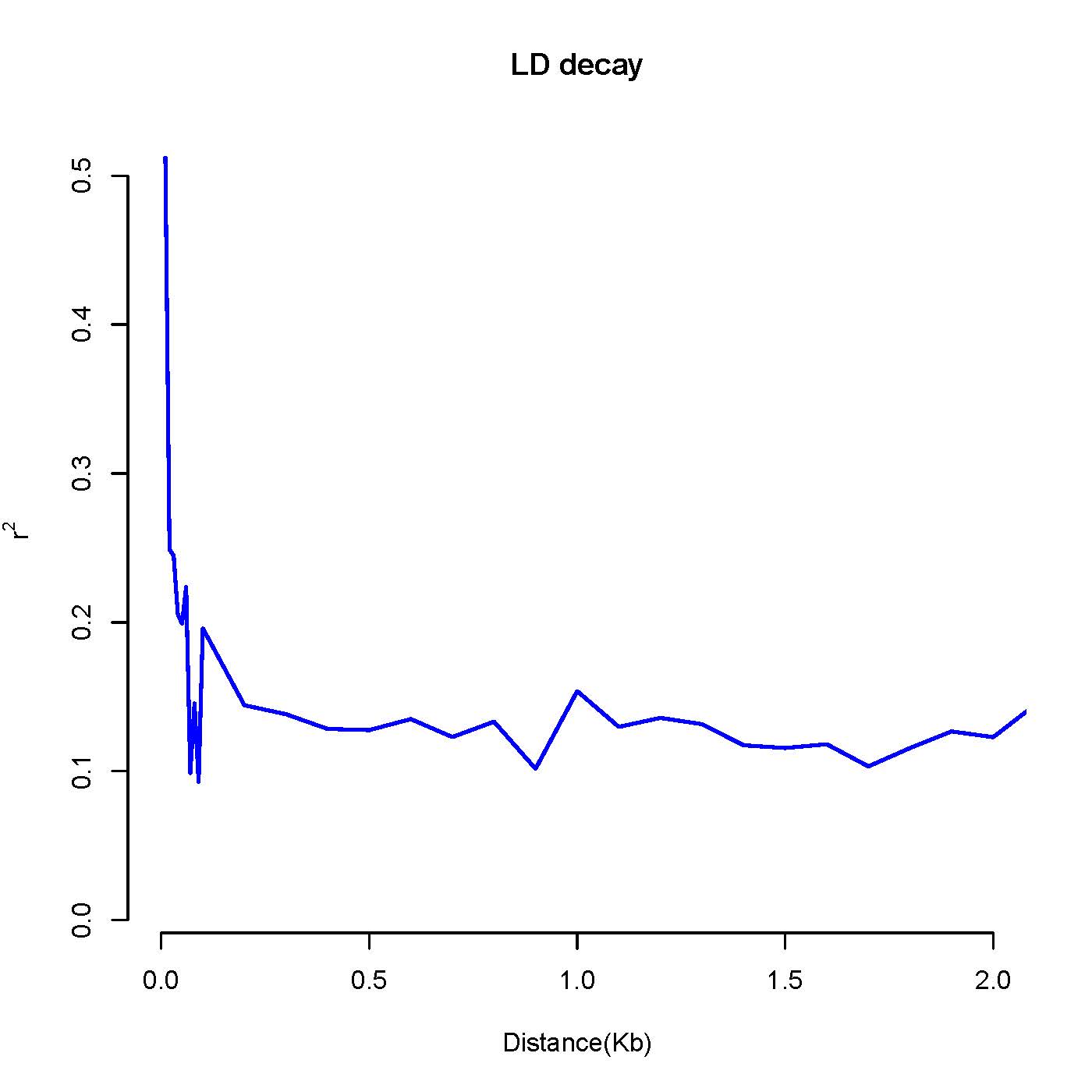

Supplement: Supplementary file 1 — Supplementary material 1 (JPG 59.9 kb) [file 41598_2026_47716_MOESM1_ESM.jpg]

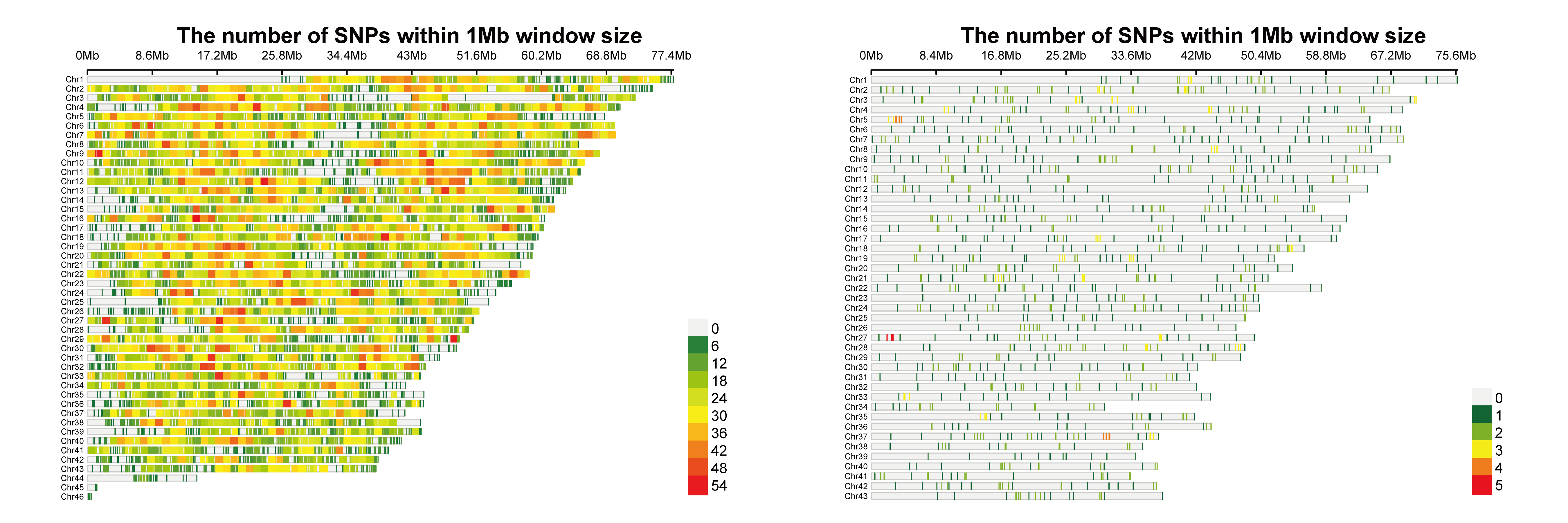

Supplement: Supplementary file 2 — Supplementary material 2 (PNG 459.3 kb) [file 41598_2026_47716_MOESM2_ESM.png]
